# Exploring the functional diversity and metabolic activities of the human gut microbiome in Thai adults in response to a prebiotic diet

**DOI:** 10.1128/spectrum.01599-24

**Published:** 2024-12-13

**Authors:** Amornthep Kingkaw, Preecha Patumcharoenpol, Narissara Suratannon, Massalin Nakphaichit, Sittiruk Roytrakul, Wanwipa Vongsangnak

**Affiliations:** 1 Interdisciplinary Graduate Program in Bioscience, Faculty of Science, Kasetsart University, Bangkok, Thailand; 2 Pediatric Allergy & Clinical Immunology Research Unit, Division of Allergy and Immunology, Department of Pediatrics, Faculty of Medicine, Chulalongkorn University, King Chulalongkorn Memorial Hospital, the Thai Red Cross Society, Bangkok, Thailand; 3 Department of Biotechnology, Faculty of Agro-Industry, Kasetsart University, Bangkok, Thailand; 4 Functional Proteomics Technology Laboratory, National Center for Genetic Engineering and Biotechnology, National Science and Technology Development Agency, Pathum Thani, Thailand; 5 Department of Zoology, Faculty of Sciences, Kasetsart University, Bangkok, Thailand; 6 Omics Center for Agriculture, Bioresources, Food, and Health, Kasetsart University (OmiKU), Bangkok, Thailand; University of Nebraska-Lincoln, Lincoln, Nebraska, USA

**Keywords:** copra meal hydrolysate, human gut microbiome, metaproteomics, gut metabolism, functional and metabolic activity

## Abstract

Exploring dietary methods to alter microbial communities and metabolic functions is becoming an increasingly fascinating strategy for improving health. Copra meal hydrolysate (CMH) is alternatively used as a gut health supplement. However, the functional diversity and metabolic activities in gut microbiome in relation to CMH treatment remain largely unknown. Therefore, this study aimed to identify key predominant groups of bacterial species toward diversified metabolic functions, activities, and routes using metaproteomics. As a result, the integrative analysis of metaproteomic data revealed that seven key families across 11 dominant gut bacterial species were concerted. Consistently, across 76,206 proteins assigned to the metabolism of the 255,964 annotated proteins, short-chain fatty acid (SCFA) biosynthesis, lipopolysaccharide (LPS) biosynthesis, and bile acid (BA) metabolism were positively associated with CMH. Further identification of cooperative metabolic routes promisingly highlighted the importance of glycolysis/gluconeogenesis, tricarboxylic acid (TCA) cycle, inositol phosphate metabolism, steroid hormone biosynthesis, O-antigen repeat unit biosynthesis, and chloroalkane and chloroalkene degradation. This work presents an initial study of metaproteomics associated with prebiotic diet in a Thai population-based cohort in a developing Southeast Asian country.

**IMPORTANCE:**

Studies primarily focused on the impact of CMH on gastrointestinal symptoms and gut microbial compositions. However, as the field moves toward understanding the relationship between microbiome and diet in relation to gut health, it is critical to evaluate how changes in metabolic activities relate to cooperative metabolic routes in the gut microbiome for promoting human health. Through the use of metaproteomics, our findings highlighted the key predominant groups of bacterial species, potential proteins, and their metabolic routes involved in gut metabolism. This study provides comprehensive insights into the fundamental relationship between microbiome and dietary supplements and suggests that metaproteomics is a powerful method for monitoring metabolic functions, activities, and routes in the gut microbiome.

## INTRODUCTION

The gastrointestinal (GI) tract harbors a dynamic and complex community of gut microbiomes that plays a crucial role in human health (1). Several factors, such as host genetics, diet, lifestyle, medication, and environment, exert influence on the gut microbiome. Moreover, gut microbial compositions can be modulated by the consumption of specific nutritional substances, such as prebiotics (2, 3). One dietary strategy for modulating the gut microbiome is the utilization of prebiotics, which beneficially affect the host by selectively stimulating the growth and/or activity of the microbiome in the GI tract (3). Additionally, prebiotics can alter the microbial communities and their functions, providing energy and maintaining gut homeostasis (4, 5).

Manno-oligosaccharides (MOS) are prebiotics composed of a linear chain of mannose, derived from mannan-rich plants, such as copra meal, a by-product of the oil extraction from dried coconut kernels. In Thailand, an annual production of 25 million metric tons of copra meal is reported (6). Copra meal hydrolysate (CMH) is obtained as a source for subsequent MOS production through enzymatic hydrolysis using β-mannanase. CMH is stable under the human GI tract conditions, particularly in the small intestine, and readily fermented by *Lactobacilli* and *Bifidobacteria* (7). Very recently, the impact of CMH on gastrointestinal symptoms and gut microbiome was revealed to have a positive relationship with gut health using integrative metagenomics (e.g., 16S rRNA gene and whole metagenome shotgun [WMGS] sequencing) (8, 9). Currently, metagenomics has rapidly become a routinely used method for characterizing the functional potential of diversities, communities, and functions of gut microbiome. Nevertheless, this method does not possess the capability to directly unveil the functional diversity and activities of microbial communities.

To overcome this challenge, metaproteomics has recently emerged as an alternative approach; it can identify and quantify proteins from microbial communities at a large scale, providing direct insights into the functional diversity and activities of microbial communities at the species level. Recently, there have been a number of studies investigating the relationship between gut microbiome and several factors, that is, sex, age, disease, diet, and different treatments through metaproteomic approach (10). Despite earlier studies of the effects of CMH on the gut microbiome, however, the metabolic diversity and activities of microbial communities in response to prebiotic diet remain largely unknown. Therefore, this study is proposed to investigate metabolic diversity and activities of microbial communities in response to prebiotic diet, for example, CMH with an overall aim at identifying key predominant groups of bacterial species, metabolic functions, and their routes using metaproteomics. Initially, fecal samples were obtained from Thai adults with different treatments, that is, baseline, placebo, or CMH. Extracted microbial proteins obtained from fecal samples were then quantified and identified using liquid chromatography-tandem mass spectrometry (LC-MS/MS). Thereafter, the data were processed with different bioinformatics and systems biology tools, and databases for diversities, taxonomic profiles, and metabolic functions of the gut microbiome. The differentially expressed proteins (DEPs) and their metabolic functions were mapped and then explored. In the end, the key predominant groups of bacterial species together with metabolic functions of DEPs and their routes under cooperative gut microbiome networks were targeted in response to CMH. This study serves as a scaffold for monitoring metabolic alterations in gut microbiome in Thai and Asian cohort studies.

## RESULTS

### Assessment of metaproteomic data from the gut microbiome of Thai adults

An analysis of metaproteomic-based gut microbiome from 20 fecal samples of Thai adults yielded a total of 530,757 mass spectral counts. These counts were assigned to the following four groups: baseline placebo (bPB), baseline CMH (bCMH), treatment with placebo (tPB), and treatment with CMH (tCMH), with respective spectral counts of 122,783, 148,630, 126,139, and 133,205. The mapping of unique peptide counts revealed a total of 880,807, distributed as follows: 197,659 in bPB, 219,721 in bCMH, 224,460 in tPB, and 238,967 in tCMH (Table S1.1). Based on the spectral library and proteomic resources, 255,964 annotated proteins were selected from a total of 262,416 proteins for further analysis. The summarized results are presented in Table 1.

**TABLE 1:** Assigned spectral counts, unique peptide counts, and number of proteins^*b*^

Protein annotation	Number of proteins	Total spectral counts (Mean ± SD)				Total unique peptide counts (Mean ± SD)			
		bPB	bCMH	tPB	tCMH	bPB	bCMH	tPB	tCMH
Annotated proteins^*a*^	255,964	119,781	145,168	123,264	130,118	192,759	214,286	219,208	233,480
Unannotated proteins	6,452	3,002	3,462	2,875	3,087	4,900	5,435	5,252	5,487
Total	262,416	122,783	148,630	126,139	133,205	197,659	219,721	224,460	238,967
		12,278.30 ± 3,023.17	14,863.00 ± 2,332.96	12,613.90 ± 1,830.08	13,320.50 ± 2,120.85	19,765.90 ± 2,943.82	21,972.10 ± 2,908.68	22,446.00 ± 3,541.86	23,896.70 ± 1,989.81

^*a*^ Annotated protein is based on a protein ID with assigned function from the UniProt database.

^*b*^ bPB, bCMH, tPB, tCMH, and SD represent baseline placebo, baseline CMH, treatment with placebo, treatment with CMH, and standard deviation, respectively.

### Assigning protein functional diversity of the gut microbiome of Thai adults

**Fig 1:**
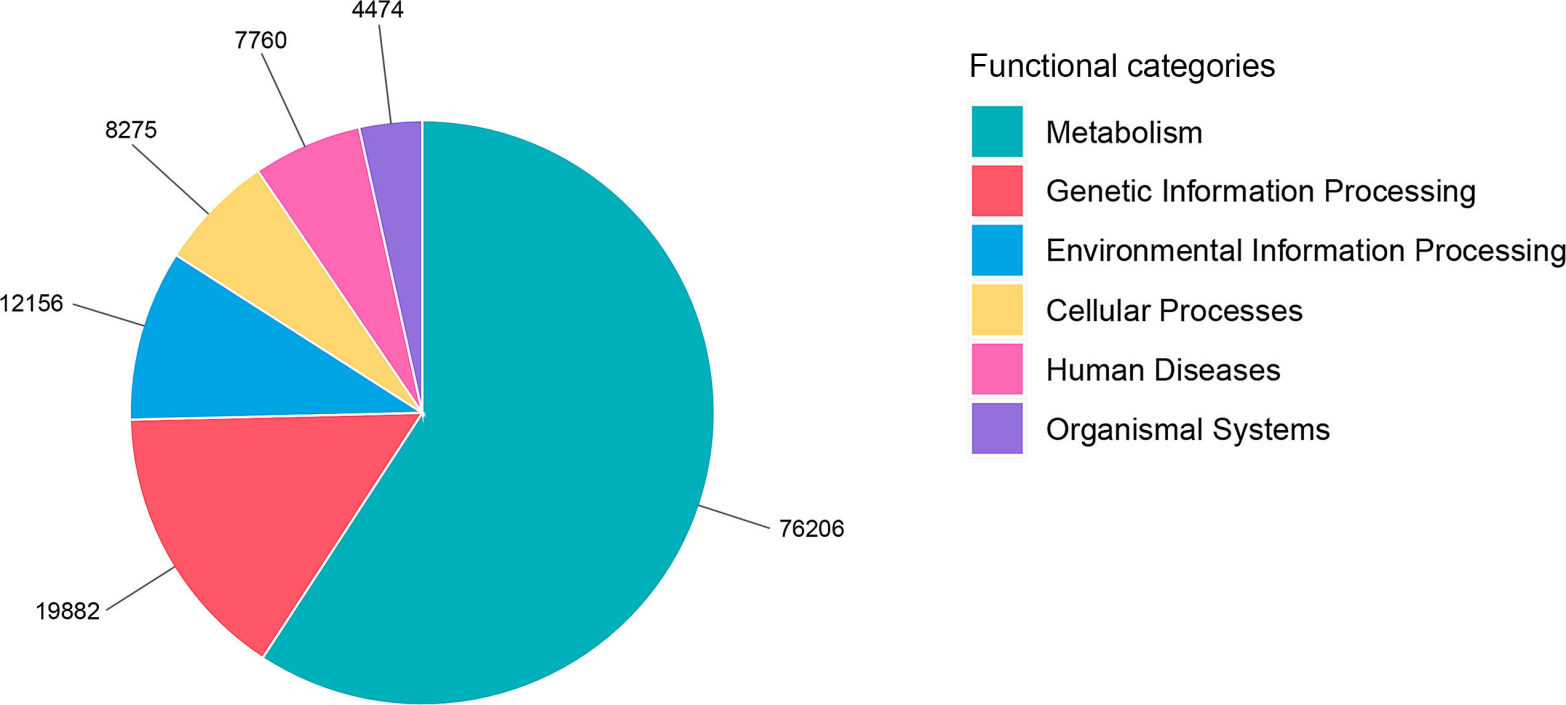
Functional diversity of proteins in the gut microbiome of Thai adults spans six main categories.

A total of 255,964 annotated proteins from the assessed metaproteomic data were assigned functions according to the Kyoto Encyclopedia of Genes and Genomes (KEGG) database (11). These proteins were categorized into six main functional groups, with metabolism being the largest category, encompassing 76,206 proteins. This was followed by genetic information processing (19,882 proteins), environmental information processing, cellular processes (8,275 proteins), human diseases (7,760 proteins), and organismal systems (4,474 proteins), as shown in Fig. 1; Table S1.2. Within the metabolic category, the functions of the proteins were further detailed: carbohydrate metabolism (20,843 proteins), energy metabolism (6,599 proteins), lipid metabolism (4,567 proteins), nucleotide metabolism (5,390 proteins), amino acid metabolism (12,800 proteins), metabolism of other amino acids (3,212 proteins), glycan biosynthesis and metabolism (8,216 proteins), metabolism of cofactors and vitamins (7,479 proteins), metabolism of terpenoids and polyketides (1,775 proteins), biosynthesis of other secondary metabolites (3,571 proteins), and xenobiotics biodegradation and metabolism (1,754 proteins) (Table S1.2). Based on KEGG database, there is no significant difference in microbial protein between the CMH and PB groups at baseline (Table S1.3).

### Investigating the taxonomy, diversity, and composition of microbial communities in relation to a prebiotic diet

**Fig 2:**
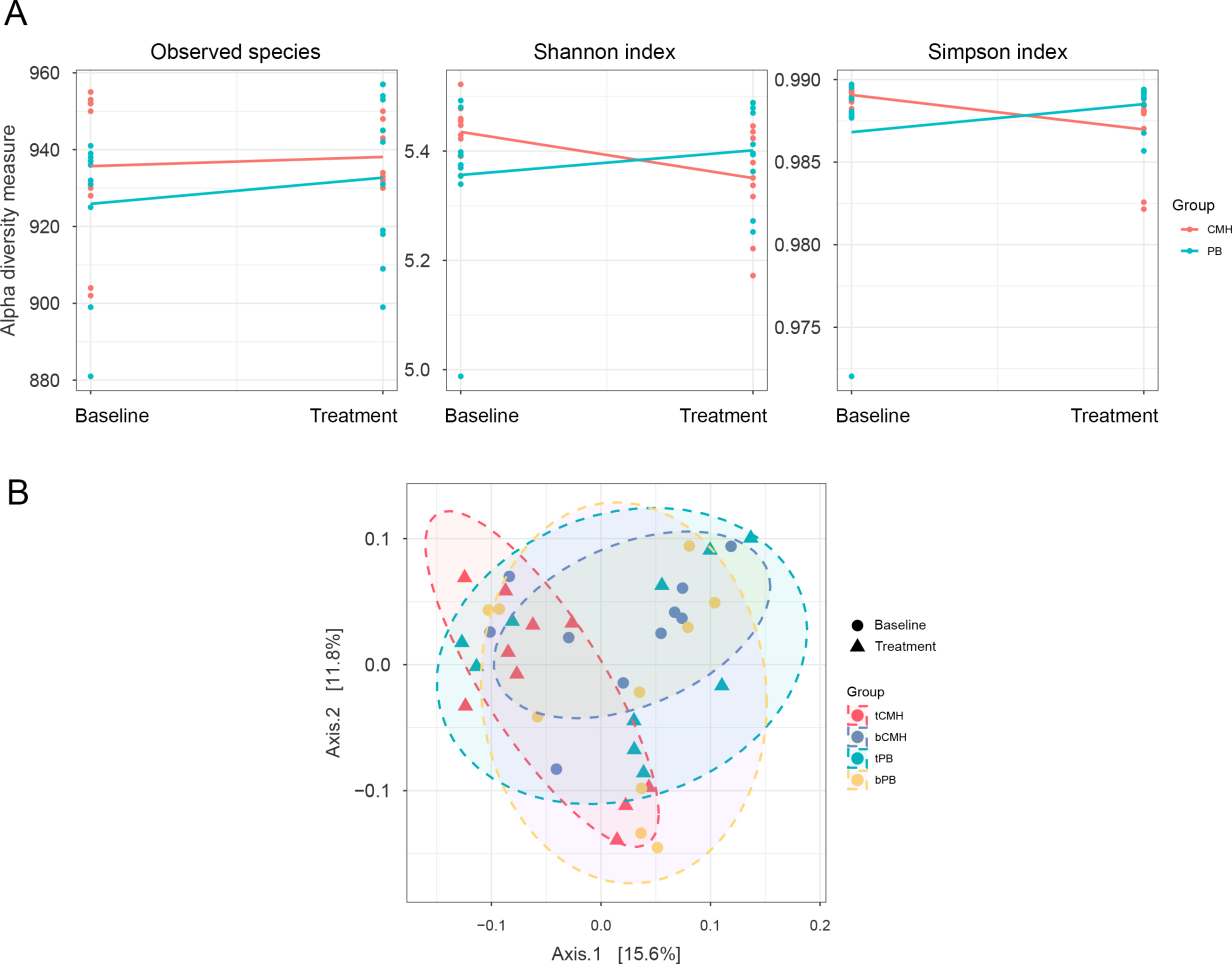
Comparison of gut microbial diversity between the CMH and PB groups. (**A**) Dot plots with regression lines depicting alpha diversity (i.e. observed species, Shannon index, and Simpson index). (**B**) PCoA plot of beta diversity based on Bray-Curtis distances across bPB, bCMH, tPB, and tCMH. Note: bPB represents baseline placebo, bCMH represents baseline CMH, tPB represents treatment with placebo, and tCMH represents treatment with CMH.

To preliminarily investigate the taxonomy and diversity of microbial community-assigned protein functions in relation to a prebiotic diet, alpha and beta diversity analyses were conducted for the CMH and PB groups. As anticipated, the alpha diversity indices, including observed species, Shannon index, and Simpson index, showed similar results between the CMH and PB groups, as illustrated in Fig. 2A. Linear modeling revealed no significant difference in microbial diversity based on observed species. Interestingly, the Shannon index indicated a statistically significant difference in microbial diversity, whereas the Simpson index exhibited a slightly different trend (Table S1.4). Considering beta diversity, a principal coordinate analysis (PCoA) plot based on Bray-Curtis distance using ADONIS2 function is presented in Fig. 2B. The results demonstrate a significant shift in microbial diversity patterns between the CMH and PB groups (ADONIS2 analysis: R² = 0.039; *P*-value = 0.038) (Table S1.5). These findings suggest that treatment with CMH impacts gut microbial diversity.

**Fig 3:**
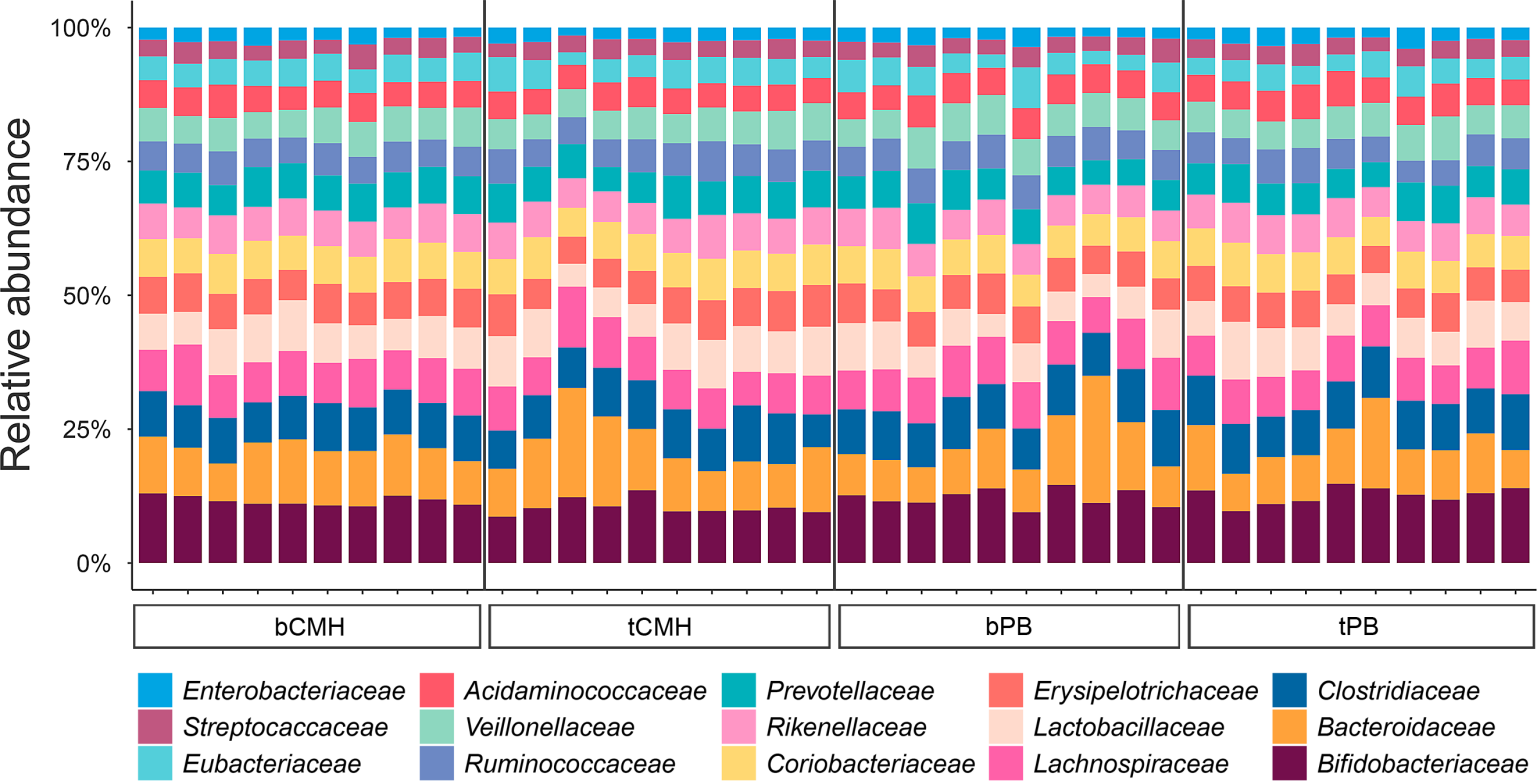
Comparison of microbial composition profiles between CMH and PB groups. Note: Stacked column illustrates the relative abundance of bacterial families across the four groups: bPB, bCMH, tPB, and tCMH.

**Fig 4:**
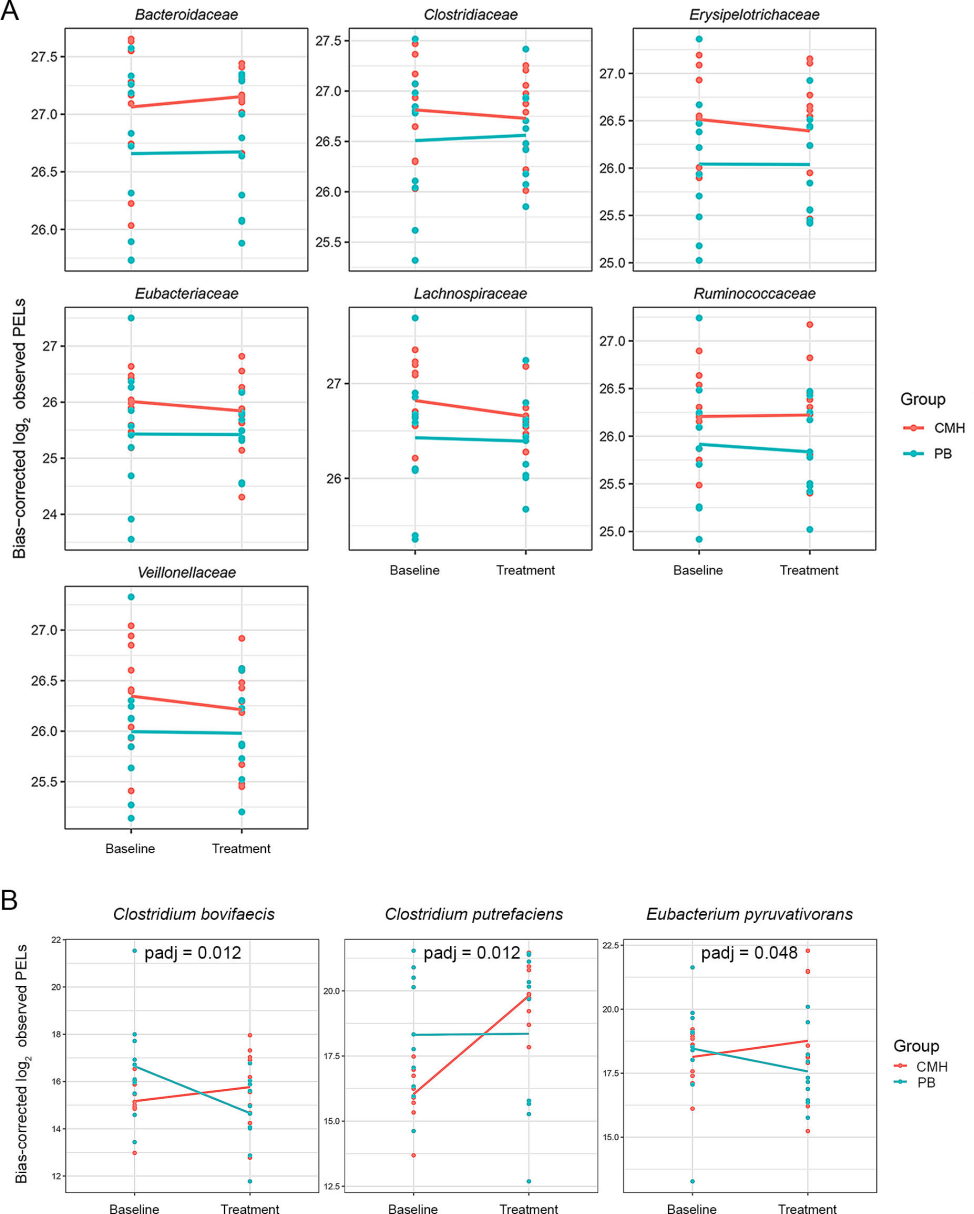
Comparison of bacterial families and species between CMH and PB groups. (**A**) Seven bacterial families associated with CMH. (**B**) Three significant species between CMH and PB groups (*P* adj < 0.05). Note: Dot plots with regression lines depict the log_2_ observed abundance corrected by DESeq2.

Regarding microbial composition profiles, the relative abundance of 15 bacterial families was comparable between the CMH and PB groups (see Materials and Methods) as shown in Fig. 3. Notably, *Bacteroidaceae* exhibited the highest relative abundance in the CMH and PB groups, accounting for 20.41% and 23.74%, respectively. Interestingly, seven predominant bacterial families, that is, *Bacteroidaceae*, *Clostridiaceae*, *Erysipelotrichaceae*, *Eubacteriaceae*, *Lachnospiraceae*, *Ruminococcaceae*, and *Veillonellaceae* were positively associated with CMH, as illustrated in Fig. 4A; Table S1.6. Notably, three significant species,that is, *Clostridium bovifaecis*, *Clostridium putrefaciens*, and *Eubacterium pyruvativorans,* showed a statistical difference between the CMH and PB groups (*P* adj <0.05), as illustrated in Fig. 4B; Table S1.7.

### Exploring metabolic functional activities in association with prebiotic diet through differentially expressed protein analysis

**Fig 5:**
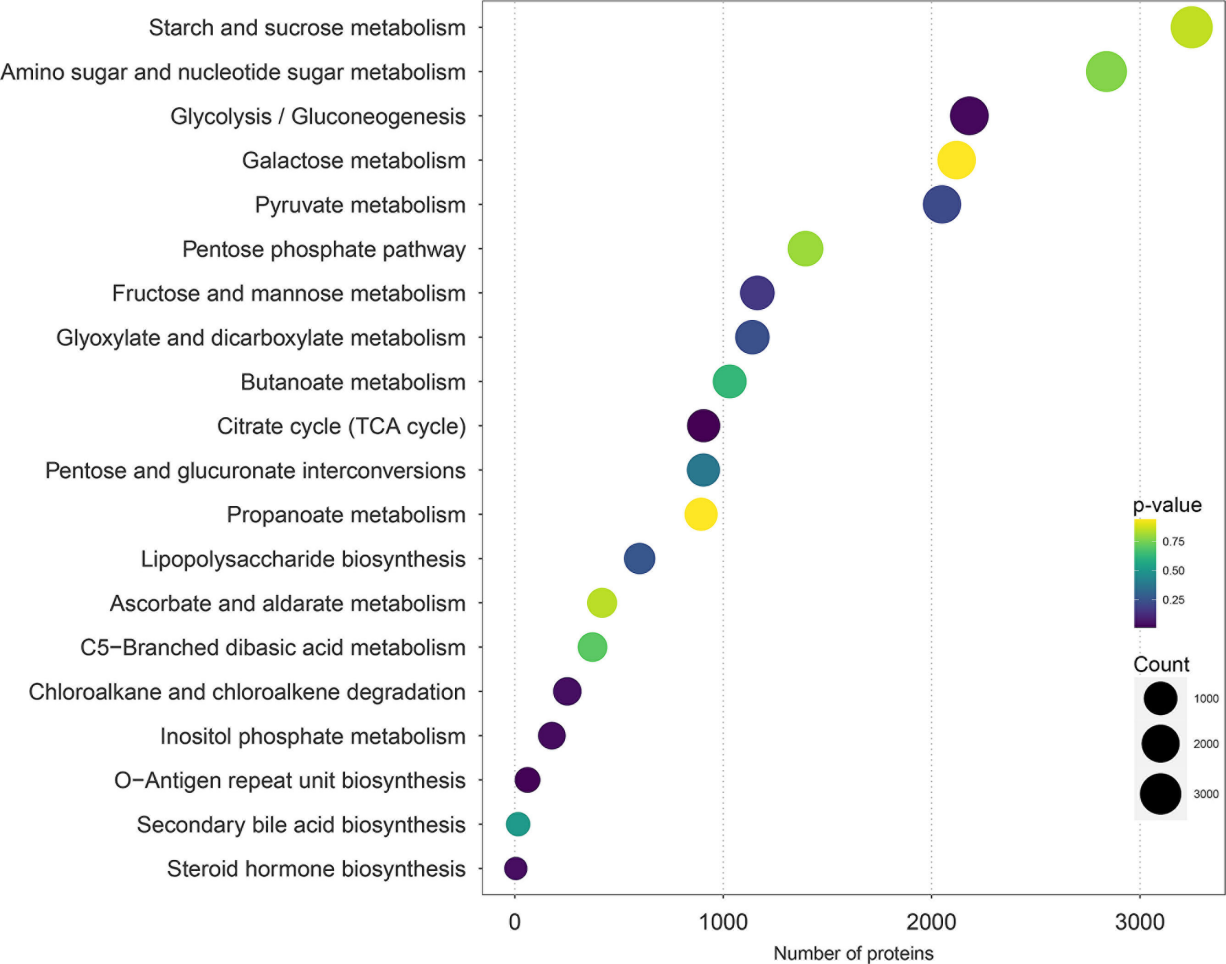
Top 20 metabolic pathways with a high number of protein functional activities.

Of 76,206 proteins associated with metabolism, the top 20 pathways of 146 metabolic pathways with a high number of protein functional activities were identified (Fig. 5; Table S1.2). They were namely starch and sucrose metabolism (3,248 proteins), amino sugar and nucleotide sugar metabolism (2,839 proteins), glycolysis/gluconeogenesis (2,181 proteins), galactose metabolism (2,120 proteins), pyruvate metabolism (2,050 proteins), pentose phosphate pathway (1,395 proteins), fructose and mannose metabolism (1,164 proteins), glyoxylate and dicarboxylate metabolism (1,140 proteins), butanoate metabolism (1,031 proteins), citrate cycle (TCA cycle) (906 proteins), pentose and glucuronate interconversions (905 proteins), propanoate metabolism (894 proteins), lipopolysaccharide biosynthesis (599 proteins), ascorbate and aldarate metabolism (419 proteins), C5-branched dibasic acid metabolism (373 proteins), chloroalkane and chloroalkene degradation (252 proteins), inositol phosphate metabolism (178 proteins), O-antigen repeat unit biosynthesis (61 proteins), secondary bile acid biosynthesis (16 proteins), and steroid hormone biosynthesis (5 proteins).

After protein functional categories, pathway and functional enrichment analysis was performed based on DEPs. Interestingly, six enriched metabolic pathways were highlighted, including the TCA cycle, O-antigen repeat unit biosynthesis, glycolysis/gluconeogenesis, inositol phosphate metabolism, steroid hormone biosynthesis, and chloroalkane and chloroalkene degradation. (Fig. 5; Table S1.8). Moreover, observably 453 significant proteins of 76,206 proteins were identified under *P* adj <0.1 (Table S1.9). These are essential for nutrient processing, immune modulation, host metabolism regulation, gut integrity maintenance, and ecosystem balance. Understanding these pathways enhances our ability to manipulate the microbiome for health and therapeutic benefits. Focusing on the top enriched significant pathways, 127 KO IDs were identified, and five targeted KO IDs, that is, K00627, K06606, K12343, K02851, and K02586, were significant (*P* < 0.05) (Table S1.10). Across targeted KO IDs against 453 significant proteins, nine key proteins were positively associated with CMH. The results are listed in Table 2; Table S1.11.

**TABLE 2:** List of key proteins associated with metabolic functional activities which significantly enriched in CMH^*a*^

Protein ID	Protein name	Category	KO ID	*P* adj
G5H8J6	Dihydrolipoamide acetyltransferase component of pyruvate dehydrogenase complex	Glycolysis/Gluconeogenesis, Citrate cycle (TCA cycle)	K00627	0.098107
A0A1H0BQ85	Dihydrolipoamide acetyltransferase component of pyruvate dehydrogenase complex	Glycolysis/Gluconeogenesis, Citrate cycle (TCA cycle)	K00627	0.051396
A0A6N9PAY8	Sugar phosphate isomerase/epimerase	Inositol phosphate metabolism	K06606	0.098107
A0A3B9CP90	3-oxo-5-alpha-steroid 4-dehydrogenase	Steroid hormone biosynthesis	K12343	0.098107
A0A8B3C6B7	Undecaprenyl/decaprenyl-phosphate alpha-N-acetylglucosaminyl 1-phosphate transferase	O-Antigen repeat unit biosynthesis	K02851	0.098107
R7JPN7	UDP-N-acetylmuramyl pentapeptide phosphotransferase/UDP-N-acetylglucosamine-1-phosphate transferase	O-Antigen repeat unit biosynthesis	K02851	0.098107
F2BWI1	UDP-N-acetylglucosamine: undecaprenyl-P N-acetylglucosaminyl 1 P transferase	O-Antigen repeat unit biosynthesis	K02851	0.098107
R6I712	Oxidoreductase nitrogenase component 1	Chloroalkane and chloroalkene degradation	K02586	0.098107
A0A170NNM0	Nitrogenase protein alpha chain	Chloroalkane and chloroalkene degradation	K02586	0.098107

^*a*^ Protein ID was selected under *P* adj <0.1.

**Fig 6:**
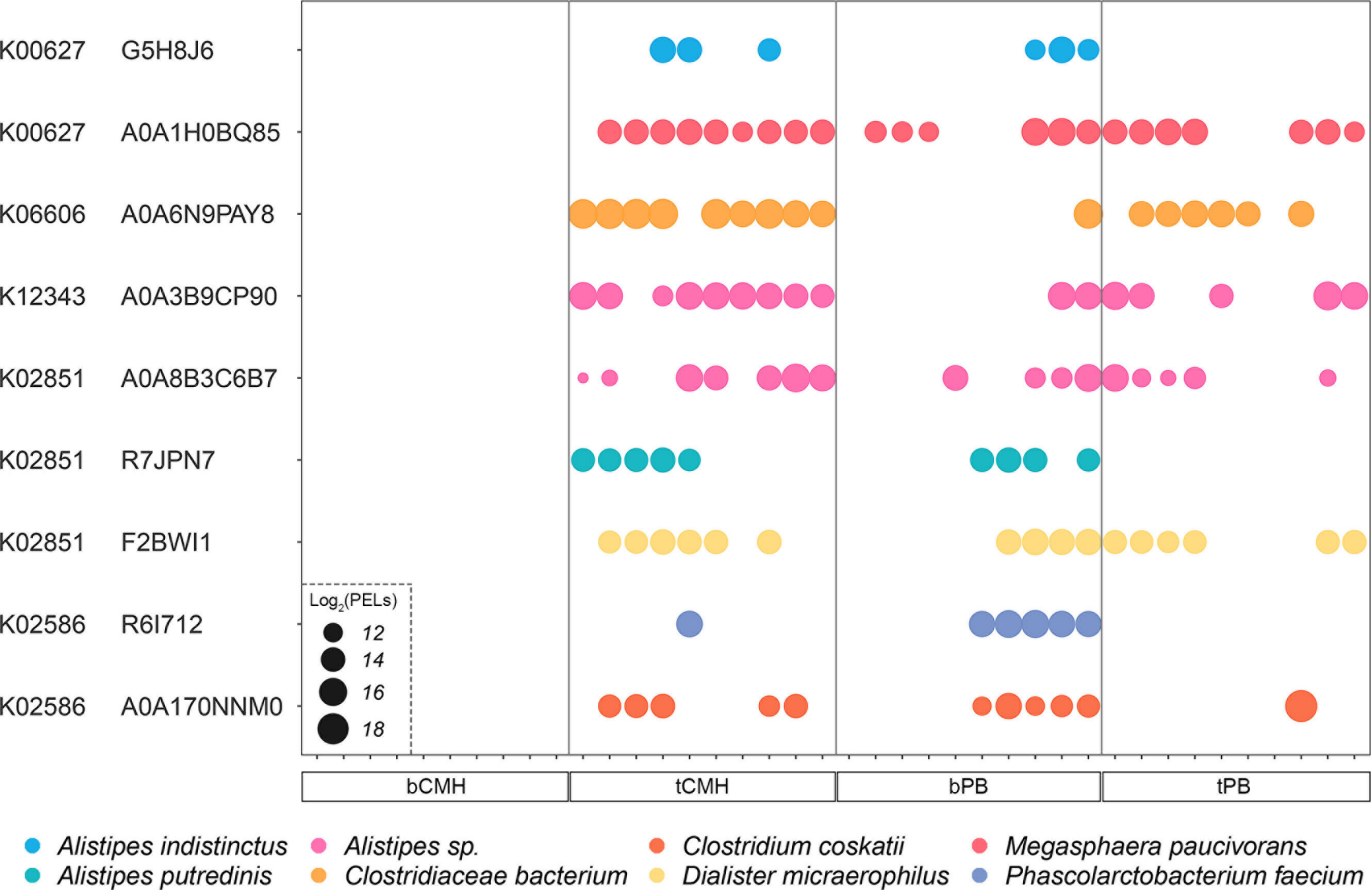
Bubble plot represents the significant proteins across different bacterial species underlying targeted KO IDs. Note: bPB represents baseline placebo, bCMH represents baseline CMH, tPB represents treatment with placebo, and tCMH represents treatment with CMH. No bubbles mean no protein expression (zero expression).

Elaborately, we identified the dihydrolipoamide acetyltransferase component of pyruvate dehydrogenase complex (G5H8J6 and A0A1H0BQ85, K00627) involved in glycolysis/gluconeogenesis and citrate cycle (TCA cycle) from *Alistipes indistinctus* and *Megasphaera paucivorans*; sugar phosphate isomerase/epimerase (A0A6N9PAY8, K06606) involved in inositol phosphate metabolism from *Clostridiaceae bacterium*; 3-oxo-5-alpha-steroid 4-dehydrogenase (A0A3B9CP90, K12343) involved in steroid hormone biosynthesis from *Alistipes* sp.; undecaprenyl/decaprenyl-phosphate alpha-N-acetylglucosaminyl 1-phosphate transferase (A0A8B3C6B7, K02851) involved in O-antigen repeat unit biosynthesis from *Alistipes* sp.; UDP-N-acetylmuramyl pentapeptide phosphotransferase/UDP-N-acetylglucosamine-1-phosphate transferase (R7JPN7, K02851) involved in O-antigen repeat unit biosynthesis from *Alistipes putredinis*; UDP-N-acetylglucosamine:undecaprenyl-P N-acetylglucosaminyl 1 P transferase (F2BWI1, K02851) involved in O-antigen repeat unit biosynthesis from *Dialister micraerophilus*; oxidoreductase nitrogenase component 1 (R6I712, K02586) involved in chloroalkane and chloroalkene degradation from *Phascolarctobacterium faecium*; and nitrogenase protein alpha chain (A0A170NNM0, K02586) involved in chloroalkane and chloroalkene degradation from *Clostridium coskatii*. Remarkably, these eight bacterial species were manually curated and associated with short-chain fatty acids (SCFAs) as shown in Fig. 6.

### Identifying cooperative metabolic routes analysis in association with prebiotic diet

**Fig 7:**
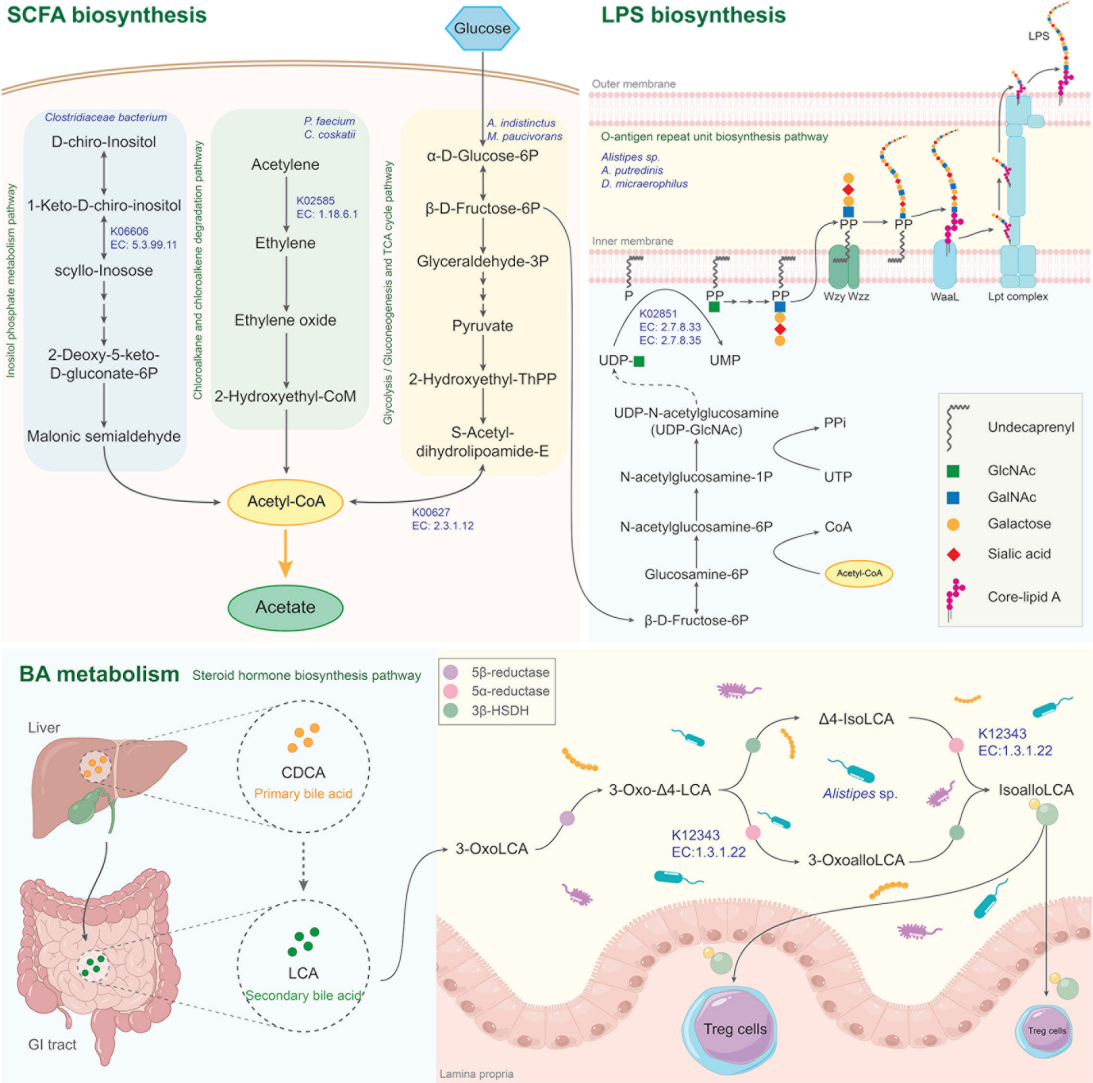
The concerted microbial communities and associated metabolic activities involved in SCFA biosynthesis, LPS biosynthesis, and BA metabolism pathways.

To identify the cooperative metabolic routes related to human gut, the key predominant bacterial species and crucial metabolic activities were integrated upon treatment with prebiotic diet, that is, CMH. Hereby, the cooperative metabolic routes are identified as shown in Fig. 7. Promisingly, cooperative routes of SCFAs biosynthesis, lipopolysaccharide (LPS) biosynthesis, and bile acid (BA) metabolism were communicated through eleven predominant species (*A. indistinctus*, *A. putredinis*, *Alistipes* sp., *Clostridiaceae bacterium*, *C. bovifaecis*, *C. coskatii*, *C. putrefaciens*, *D. micraerophilus*, *E. pyruvativorans*, *M. paucivorans,* and *P. faecium*).

Regarding SCFAs biosynthesis, the three metabolic pathways were highlighted, for example, inositol phosphate metabolic pathway, chloroalkane and chloroalkene degradation pathway, as well as glycolysis/gluconeogenesis and TCA cycle pathway, which are responsible for acetate production. For inositol phosphate metabolic pathway, we found key enzyme 2-keto-myo-inositol isomerase (K06606, EC:5.3.99.11) identified in *Clostridiaceae bacterium*, which is capable of converting 1-keto-D-chiro-inositol to scyllo-inosose. This is important for generating key precursors like malonate semialdehyde and glyceraldehyde 3-phosphate for acetyl-CoA. As acetyl-CoA formation, we also found key enzyme (K02586, EC: 1.18.6.1) in chloroalkane and chloroalkene degradation pathway, which is capable of converting acetylene to ethylene associating with nitrogenase molybdenum-iron protein alpha chain in both *P. faecium* and *C. coskatii*. This study shows that acetyl-CoA is a key precursor for further acetate formation.

In glycolysis/gluconeogenesis and TCA cycle pathway, the key enzyme pyruvate dehydrogenase, dihydrolipoamide acetyltransferase (K00627, EC:2.3.1.12) across *A. indistinctus* and *M. paucivorans,* was found. This enzyme is a second component of the pyruvate dehydrogenase complex, responsible for transferring the acetyl group to coenzyme A (CoA) during the conversion of pyruvate into acetyl CoA (12).

Focusing on LPS biosynthesis, O-antigen repeat unit biosynthetic pathway is also illustrated (Fig. 7). There was a key enzyme, that is, UDP-GlcNAc: undecaprenyl-phosphate/decaprenyl-phosphate GlcNAc-1-phosphate transferase (K02851, EC: 2.7.8.33, EC: 2.7.8.35), across *Alistipes* sp. *A. putredinis* and *D. micraerophilus*. This enzyme transfers GlcNAc-1-P from UDP-GlcNAc to membrane-bound P-Und, forming GlcNAc-PP-Und. Subsequently, it is extended to form the repeating unit by sequential addition of sugars by specific glycosyltransferases (GTs) (13, 14).

Considering the BA metabolism, the steroid hormone biosynthetic pathway involved in gut microbiome-shaped BAs is also depicted (Fig. 7). A key enzyme involved in steroid dehydrogenase: 3-oxo-5α-steroid 4-dehydrogenase 1 (K12343, EC:1.3.1.22) in *Alistipes* sp. This can convert a secondary BA metabolite 3-oxolithocholic acid (3-oxoLCA) to isoalloLCA. The 3-oxoLCA is converted to isoalloLCA by sequential actions of three enzymes: Δ^4^-3-oxosteroid 5β-reductase (5β-reductase), 3-oxo-5α-steroid 4-dehydrogenase (5α-reductase), and 3β-hydroxysteroid dehydrogenase (3β-HSDH), which were found in *Odoribacter* spp. and *Alistipes* spp (15).

Concerning isoalloLCA, it maintains intestinal homeostasis by promoting differentiation of naive T-cells to regulatory T cells in gut lamina propria (15, 16). IsoalloLCA was also observed to inhibit the growth of *Clostridium difficile* and other gram-positive species, such as *Bifidobacterium*, *Faecalibacterium,* and *Streptococcus*. This might lower the risk of infection and contribute to maintaining intestinal homeostasis for healthy aging. The results of this study show that key predominant bacterial species are strongly linked to the utilization of prebiotic diet, for example, CMH. These findings uncover concerted microbial communities utilizing sugars (e.g., glucose or mannose) and dietary fibers (e.g., CMH) in relation to SCFAs biosynthesis, LPS biosynthesis, and BA metabolism. These results suggest that CMH evidently influences the human gut microbiome on diversities, compositions, and metabolic responses.

## DISCUSSION

The impact of CMH has been focused on gastrointestinal symptoms and gut microbial compositions. The functional diversities and activities toward metabolic routes of microbial communities in response to prebiotic diet, for example, CMH, were mainly studied. Regarding microbial diversities, the results revealed no significant difference in gut microbial richness and alpha diversity indices when treated with CMH (8, 9). For beta-diversity indices, our results demonstrated a distinct microbial community composition after treatment with CMH. These suggest significant alterations in microbial composition structure by prebiotic diet uptake.

In terms of microbial taxonomic profiles contributing to these diversity patterns, noticeably, CMH increases the abundance of bacterial families, that is, *Bacteroidaceae, Clostridiaceae, Erysipelotrichaceae, Eubacteriaceae, Lachnospiraceae, Ruminococcaceae*, and *Veillonellaceae*. This result aligns with previous studies finding that treatment with CMH could enhance the abundance of the gut microbiome, including *Acidaminococcaceae*, *Coriobacteriaceae*, *Erysipelotrichaceae*, R*uminococcaceae*, and *Veillonellaceae* (9). In addition, the results are consistent with those of earlier reports that these bacterial families contain enzymes involved in the degradation of hemicellulose (e.g., xylan, mannan, and galactomannan), cellulose, or dietary fiber (17–20). Interestingly, significant differences were observed in the gut microbial composition at the species level, that is, *C. bovifaecis, C. putrefaciens*, and *E. pyruvativorans* in relation to CMH. Consistent with prior information, members of the *Clostridium* exhibit both fermentative and electrogenic activity, enabling them to degrade a wide range of substrates, such as cellulose (21). Additionally, *Clostridium* has also been reported to ferment glycerol, xylose, or pentosans into SCFAs, for example, acetate production (22). Moreover, *C. bovifaecis* and *C. putrefaciens* belonging to the *Clostridium* are generally involved in SCFA production. Meanwhile, *E. pyruvativorans*, belonging to the *Eubacterium*, is a non-saccharolytic anaerobe that fermented pyruvate and amino acids known as SCFA production (i.e., caproate and valerate) and BA metabolism (23–26).

Furthermore, our findings are also consistent with those of Rowland et al. (27), demonstrating various microbially mediated modifications at the steroid nucleus that led to secondary bile acids. *Clostridium* and *Eubacterium* have the capability to transform chenodeoxycholic acid (CDCA) and cholic acid (CA) into the secondary bile acids lithocholic acid (LCA) and deoxycholic acid (DCA), respectively. These findings suggest that the analysis of microbial diversity and composition in the gut is useful for signifying the impacts of CMH interventions in humans.

A quantitative assessment was conducted of metabolic function across DEPs and enrichment analysis. An identification of enzymes involved in metabolism that were significantly enriched in CMH was of particular interest. Indeed, key enzymes and a predominant bacterial species associated with SCFA biosynthesis, LPS biosynthesis, and BA metabolism were significantly enriched in CMH (28–34).

Beyond our metaproteomics with integrated metagenomics (i.e., 16S rRNA gene sequence and WMGS data sets), this finding reveals the cooperative metabolic routes facilitated by 23 predominant bacterial species, including *A. butyriciproducens, A. hadrus, A. indistinctus, A. intestini, A. putredinis, Alistipes sp., B. dorei, B. massiliensis, B. vulgatus, C. bovifaecis, C. coskatii, C. putrefaciens, C. saccharolyticum, Clostridiaceae bacterium, D. micraerophilus, D. piger, E. coli, E. pyruvativorans, E. siraeum, M. paucivorans, P. faecium, R. hominis,* and *R. intestinalis*. These predominant bacterial species participated in potential metabolic pathways, including glycolysis/gluconeogenesis, the TCA cycle, pentose and glucuronate interconversions, propanoate metabolism, C5-branched dibasic acid metabolism, inositol phosphate metabolism, steroid hormone biosynthesis, O-antigen repeat unit biosynthesis, and chloroalkane and chloroalkene degradation. Regarding the predominant bacterial species and potential metabolic pathways, we interestingly found that the genus *Clostridium* and glycolysis/gluconeogenesis as well as TCA cycle pathways were commonly observed in both metaproteomics and metagenomics (Table S1.12). This integrative finding not only uncovers the cooperative metabolic routes underpinning gut health but also specifically identifies critical bacterial species and potential metabolic pathways that may serve as targets for therapeutic interventions, highlighting their roles in sustaining gut homeostasis and influencing host health.

In light of our study, the relationship between gut microbiome and dietary supplements is revealed to be cooperative networks within gut microbial communities, providing insight into the metabolic diversity and activities of microbial communities and the complexities of the diet-microbiome relationship.

### Conclusions

Metaproteomics-based analysis reveals functional diversity and metabolic activities of the human gut microbiome in Thai Adults. In response to prebiotic diet, for example, CMH, cooperative metabolic routes within gut microbial communities under the utilization of sugars and dietary fibers were unveiled. CMH is thus proposed to be the alternative intervention as a potential prebiotic diet for modulating and maintaining gut metabolism. This study serves as a roadmap for monitoring metabolic functional diversity and activities of gut microbiome toward its implications for human health.

## MATERIALS AND METHODS

### Participant enrollment and fecal sample collection

Twenty Thai adults residing in Bangkok and neighborhood aged between 22 and 39 years with a body mass index of 18.5–24.8 kg/m^2^ were enrolled in double-blinded, placebo-controlled trials. Regarding the recruitment process, the participants were informed at King Chulalongkorn Memorial Hospital in Bangkok, Thailand, under stringent inclusion and exclusion criteria, including considerations, such as dietary intake, age, and health status. Importantly, the participants had no history of intestinal diseases or diarrheal symptoms in the months preceding sampling, and none had a family history of colorectal cancer. Additionally, participants refrained from taking antibiotics for at least 3 months, as well as avoiding probiotics, prebiotics, and synbiotics for a minimum of 1 month before sampling. Participants with allergies to coconuts or food intolerance were excluded. The demographic characteristics of the study participants in this cohort are presented in Table 3; Table S1.13. The preparation of CMH and placebo drinks, study design, randomization and allocation concealment, and intervention were detailed according to the methodology outlined by Sathitkowitchai et al. (8).

**TABLE 3:** Demographic characteristics of study participants at baseline^*a*^

Variable	Placebo	CMH	*P-*value
Number	10	10	1.000
Age (year)	29 ± 4.32	31 ± 5.95	0.286
Weight (Kg)	54 ± 7.76	49 ± 7.31	0.198
Height (cm)	159 ± 4.29	156 ± 4.92	0.182
Body mass index	21 ± 2.30	20 ± 1.55	0.436

^*a*^ All values are expressed as mean ± SD. The *P*-values were calculated using the Wilcoxon rank-sum test.

For fecal sample collection, the participants were assigned into two groups (CMH and PB) at baseline and treatment, resulting in a total of 40 samples: bPB for 10 samples, bCMH for 10 samples, tPB for 10 samples, and tCMH for 10 samples. Notably, baseline means that they were not treated with either CMH or PB, whereas treatment means that they were subjected to CMH or PB for 21 days. Fecal samples (20 g) were immediately collected at the time of defecation and placed into a collection tube in a cooler bag. The fecal samples were stored at −80°C for further analysis.

This study was approved by the Thai Clinical Trials Registry (trial identification number: TCTR20190426003) and the Ethics Committee of King Chulalongkorn Memorial Hospital, Bangkok, Thailand (IRB No. 388/61). All methods were performed in accordance with relevant guidelines and regulations. Written informed consent was obtained from all participants.

### Microbial protein extraction

Sample preparation was performed as previously described by Losuwannarak et al. (35). Briefly, frozen fecal samples were reconstituted in 50 mM phosphate buffer pH 7.0 and then vortexed well. After centrifugation for 10 min at 12,000 rpm to remove debris and some large particles (36), the solubilized protein remaining in the clear supernatant was collected. Total soluble protein was measured with a Lowry assay using bovine serum albumin as a standard (37). In 5 mg protein samples, disulfide bonds were reduced using 5 mM dithiothreitol in 10 mM ammonium bicarbonate at 60°C for 1 h, followed by the alkylation of sulfhydryl groups by 15 mM iodoacetamide in 10 mM ammonium bicarbonate for 45 min in the dark at room temperature. For digestion, the protein samples were mixed with sequencing-grade trypsin (ratio of 1:20) (Promega, Germany) and incubated at 37°C overnight. Prior to LC-MS/MS analysis, the digested protein (tryptic peptide) samples were dried and protonated with 0.1% formic acid before injection into the LC-MS/MS system.

### Liquid chromatography-tandem mass spectrometry analysis

LC-MS/MS was conducted as previously described in Losuwannarak et al. (35). Specifically, the tryptic peptide samples (100 ng) were injected in triplicates into an UltimateTM 3000 Nano/Capillary LC System (Thermo Scientific) coupled to a Hybrid quadrupole Q-TOF impact II (Bruker Daltonics) equipped with a Nano-captive spray ionization (CSI) source. Here, the peptides underwent an enrichment step utilizing a μ-Precolumn (300 mm i.d. × 5 mm) packed with C18 PepMap 100, 5 mm, 100 A° (Thermo Scientific) and separated on a column (75 mm I.D. × 15 cm) filled with Acclaim PepMap RSLC C18, 2 mm, 100, nanoViper (Thermo Scientific). A mobile phase of solvent A (0.1% formic acid) and solvent B (80% acetonitrile and 0.1% formic acid) were applied to the analytical column. A linear gradient ranging from 5% to 55% solvent B was utilized to elute the peptides at a constant flow rate of 0.30 mL/min for 30 min. Electrospray ionization was performed at 1.6 kV using the CaptiveSpray system. Mass spectra (MS) and MS/MS spectra were acquired in the positive ion mode at a frequency of 2 Hz, covering the range of m/z 150–2,200.

### Quantification and identification of microbial proteins and database search

For the quantification of proteins, MaxQuant (version 2.1.4.0) was used to quantify individual samples and submit their MS/MS spectra to match with the UniProt bacterial databases by using the Andromeda search engine (38). The parameters for label-free quantitation and identification with MaxQuant were (ⅰ) a maximum of two missed cleavages, (ⅱ) mass tolerance of 0.6 Daltons for the main search, (ⅲ) trypsin as the digestion enzyme, (ⅳ) carbamidomethylation of cysteine residues as a fixed modification, and (ⅴ) oxidation of methionine and acetylation of the protein N-terminus as variable modifications. The significance threshold for protein identification was established with a *P*-value < 0.05 and a false discovery rate (FDR) of 1%. For searches in FASTA files, a protein database of 15 candidate bacterial families*—Acidaminococcaceae*, *Bacteroidaceae*, *Bifidobacteriaceae*, *Clostridiaceae*, *Coriobacteriaceae*, *Enterobacteriaceae*, *Erysipelotrichaceae*, *Eubacteriaceae*, *Lachnospiraceae*, *Lactobacillaceae*, *Prevotellaceae*, *Rikenellaceae*, *Ruminococcaceae*, *Streptococcaceae*, and *Veillonellaceae,* selected from earlier reports of gut microbiome data from Thailand (9, 39, 40)—was downloaded from UniProt. Database with potential contaminants included in MaxQuant was automatically added. The MaxQuant ProteinGroups.txt file was subsequently obtained in conjunction with the use of Perseus software (version 1.6.6.0) for importing peptide sequences into the metaproteome data set (41). Exact peptides for which a unique protein sequence was matched to a single bacterial strain were classified as bacterial strain-specific sequences for taxonomic classification (42, 43). The remaining peptides for which a unique protein sequence was not matched to a single bacterial strain were discarded. The protein sequences assigned with protein IDs with known/putative functions from the UniProt bacterial database were denoted as annotated proteins. In contrast, the protein sequences assigned an ID corresponding to a hypothetical protein/uncharacterized protein were designated as unannotated proteins. For quality control, the mean and standard deviation (SD) values were calculated for the total spectral counts and total unique peptide counts. Maximum peptide intensities were selected, providing the protein expression levels (PELs). For further functional diversity, annotated proteins were assigned and categorized using the KEGG database.

### Microbial diversity, taxonomy, and metabolic functional analysis

Metaproteome data were analyzed for microbial diversity, community composition, and metabolic functional analysis for both CMH and PB groups. Alpha- and beta-diversity analyses were conducted using a vegan package in the R program (version 2.5–6). For alpha-diversity, the observed species, Shannon’s index, and Simpson’s index were used to calculate species richness and abundance. Beta-diversity was assessed using Bray-Curtis distances with the metaMDS function in the vegan R package (44). PCoA was performed to visually evaluate the differences in microbial community structure across sample conditions using the ggplot2 R package (45). Statistical differences in associations between diversity values and sample groups, that is, CMH and PB were calculated using linear regression for alpha-diversity and permutational multivariate analysis of variance (ADONIS) for beta-diversity.

Concerning differential abundance of bacteria and proteins, PELs were used to infer taxonomic levels and DEPs. For microbial taxonomy according to the 15 selected bacterial families, the PELs were grouped based on taxonomic information in the sequence database and then were summed to obtain total PELs for taxa at each level (i.e., family, genus, and species) in each sample. The relative abundance was plotted using the ggplot2 package in R program. The DEPs analysis was afterward performed between CMH and PB groups using total PELs for each abundant protein. The difference in abundance of bacterial taxa and proteins was examined using negative binomial generalized linear models (DESeq2) (46). The likelihood-ratio tests (LRT) in DESeq2 model were used to determine the statistical significance of different treatments, which allows us to estimate the treatment effect on protein expression by normalizing for variations between samples, ensuring that the analysis reflected genuine biological changes. The apeglm method for log2FC shrinkage was used to account for dispersion and variation of effect size across individuals and treatments. Only bacteria and proteins with independent hypothesis weighted log2FC > 0 were regarded as positively related to CMH. Benjamini-Hochberg correction was used for multiple testing to define differentially abundant bacteria and proteins (FDR < 0.05).

For the metabolic functional annotation, the DEPs were searched against the KEGG database and then assigned to KEGG Orthology (KO) IDs using GhostKOALA (47). After the identification of KO IDs, they were categorized into main functions, subfunctions, and pathways. Statistical differences in microbial protein between CMH and PB at baseline were calculated using permutational multivariate analysis of variance (ADONIS). Additionally, pathway and functional enrichment analysis was performed using GSEA (48) in the R package piano based on DEPs between CMH and PB groups. Pathways and functions with a distinct updirectional *P*-value < 0.05 were considered significantly enriched.

### Identification of key predominant groups of bacterial species, potential proteins, and their metabolic routes in relation to prebiotic diet

To identify the key predominant groups of bacterial species, potential proteins, and associated routes, the results from microbial taxonomy and metabolic functions obtained from metaproteomic data were integrated. Subsequently, abundant bacterial species (*P* adj <0.05) and enriched KO IDs (*P* adj <0.1) were then considered. The targets of enriched KO IDs across abundant bacterial species were mapped to target metabolic pathways using the KEGG database. Upon mapping abundant bacterial species onto target metabolic pathways, the abundant species in each pathway were considered to have the ability to act together in a community as key predominant groups of bacterial species. Furthermore, manual curation and literature mining were also conducted to reveal the key predominant groups of bacterial species and a list of potential proteins as well as associated routes for generating cooperative networks within microbial communities.

## Data Availability

The mass spectrometry proteomic data have been deposited with the ProteomeXchange: PXD052903 and JPST003162.

## SUPPLEMENTAL MATERIAL

Supplemental material spectrum.01599-24-s0001.xlsx Tables S1 to S13.

ASM does not own the copyrights to Supplemental Material that may be linked to, or accessed through, an article. The authors have granted ASM a non-exclusive, world-wide license to publish the Supplemental Material files. Please contact the corresponding author directly for reuse.
